# Akinetic crisis and withdrawal syndromes: guideline “Parkinson’s disease” of the German Society of Neurology

**DOI:** 10.1007/s00415-024-12649-x

**Published:** 2024-08-27

**Authors:** Monika Pötter-Nerger, Matthias Löhle, Günter Höglinger, German Parkinson‘s Guideline Group

**Affiliations:** 1 Department of Neurology, University Medical Center Hamburg-Eppendorf, Martinistraße 52, 20246 Hamburg, Germany; 2 Department of Neurology, University Medicine Rostock, Rostock, Germany; 3 German Center for Neurodegenerative Diseases (DZNE), Rostock, Germany; 4 Department of Neurology, LMU University Hospital, Ludwig-Maximilians-Universität (LMU) München, Munich, Germany; 5 German Center for Neurodegenerative Diseases (DZNE), Munich, Germany; 6 Munich Cluster for Systems Neurology (SyNergy), Munich, Germany

**Keywords:** Akinetic crisis, Dopamine agonist withdrawal syndrome, Deep brain stimulation withdrawal syndrome, Diagnostic criteria, Therapy, German Parkinson Guideline

## Abstract

The akinetic crisis is a well-known, rare, potentially life-threatening condition in Parkinson’s disease with subacute worsening of akinesia, rigidity, fever, impaired consciousness, accompanying vegetative symptoms and transient dopa-resistance. The akinetic crisis was historically supposed to be a “withdrawal syndrome” in the sense of discontinuation of dopaminergic medication. Recently, other “withdrawal syndromes” as the specific “dopamine agonist withdrawal syndrome” or “deep brain stimulation withdrawal syndrome” have been described as emergency situations with specific subacute symptom constellations. All three conditions require immediate start of the adequate therapy to improve the prognosis. Here, the diagnostic criteria and treatment options of these three acute, severely disabling syndromes will be reported along the current guidelines of the German Parkinson Guideline Group.

## Introduction

The akinetic crisis represents a rare, acute, potentially life-threatening worsening of symptoms in patients with Parkinson’s disease (PD) with fever, pronounced akinesia, rigidity, impaired consciousness, dysphagia, accompanying vegetative symptoms, possibly myoclonus or dystonia [1, 2]. The akinetic crisis is also often referred to “Parkinson’s hyperpyrexia syndrome”, “malignant syndrome”, “acute akinesia” or “neuroleptic malignant-like syndrome”, based on historically observed similarities with psychiatric patients on high-dose neuroleptic medication who experience fever and a severe Parkinsonian syndrome.

Historically, akinetic crisis in Parkinson’s disease (PD) is considered a “withdrawal syndrome” of dopaminergic medication, in the sense of change in the regular dopaminergic medication intake, malabsorption of dopaminergic medication during diarrhea or accidental intake of anti-dopaminergic medication such as neuroleptics, although other mechanisms as dopamine-resistance during e.g. infections are also supposed to contribute. Recently, other “withdrawal syndromes” as the specific “dopamine agonist withdrawal syndrome” or “deep brain stimulation withdrawal syndromes” [3] have been described, which represent specific, own entities with slightly different symptomatology and therapeutical approaches. All acute “withdrawal syndromes” represent emergency situations in PD, which should be recognized early for immediate start of adequate therapy. Here, the evidence in terms of diagnostic criteria and treatment options of these three acute, severely disabling, specific syndromes will be reported along the current guidelines of the German Parkinson Guideline Group.

## Methodological approach

This guidance was prepared for the German Society of Neurology (DGN) in collaboration with the Austrian Society of Neurology (ÖGN) and the Swiss Neurological Society (SNG) along the guideline commission of the DGN at S2k level. A complete version of this guideline can be found on the website of the DGN (www.dgn.org) and the Arbeitsgemeinschaft wissenschaftlicher Medizinischer Gesellschaften (AWMF, https://register.awmf.org/de/leitlinien/detail/030-010, registry No. 030/010, last update: November 30th, 2023; valid until: October 24th, 2028; last access: March 30th, 2023). After an open call by the DGN of members, experts were formally accepted following an independent assessment of their conflict of interest statements (www.dgn.org). In the framework of PICO (Patient, Intervention, Comparison, and Outcome), the systematic literature search was carried out in the PubMed databases (https://pubmed.ncbi.nlm.nih.gov/; search period: from 2016-11/ 2021; language: German and English). The search terms required were structured as described in detail in the Guideline Report available online (https://register.awmf.org/de/leitlinien/detail/030-010). The strength of recommendation followed the guidelines of the Oxford Centre for Evidence-based Medicine. All recommendations were initially voted in a Delphi process. The degree of consensus was classified as “strong consensus” in the case of  > 95% consensus among the voting experts, as “consensus” in the case of  > 75–95%, as “majority agreement” in the case of  > 50–75%, and as “no majority agreement” in the case of  ≤ 50%. The final guideline was reviewed and approved by the DGN Guideline Committee and assessed with the AGREE-II Instrument. Further details are available in the methodological Appendix section of the German guideline (https://register.awmf.org/de/leitlinien/detail/030-010).

### The akinetic crisis in Parkinson’s disease

The akinetic crisis is *defined* as an acute, potentially life-threatening worsening of symptoms in patients with Parkinson’s disease. With an incidence of 0.3% Parkinson’s patients/year, it is a relatively rare event [1]. Narrower criteria for defining akinetic crisis have been proposed as acute worsening of motor UPDRS III > 20 points accompanied by transient resistance to dopaminergic medication for  > 3 days [2]. The diagnostic differentiation of an akinetic crisis from pronounced hypokinetic fluctuation is the levodopa-resistance as a key criterion for the akinetic crisis.

*Clinical symptoms* have been described in two larger cohorts [1, 4]. In a Japanese multicenter study [4] in 72 PD patients and 21 patients with Parkinsonism, high fever was one of the most frequently observed symptoms associated with motor deterioration. In another, Italian cohort, there was a deterioration of 20–31 points on the motor part of UPDRS scale with a worsening in the Hoehn & Yahr stage from 2 to 4–5 [1]. Furthermore, about half of the patients showed a disturbance of consciousness, a state of confusion, loss of appetite, dysphagia, a general feeling of illness and autonomic symptoms such as increased sweating, hypersalivation, blood pressure fluctuations and, rarely, myoclonus [4]. Other case reports included increased tremor or dystonic symptoms [5]. Observations on the clinical course revealed symptom worsening within 2–3 days, lasting for about 11 days and recovery after 4–26 days [1]. There seem to be individual predisposing risk factors such as advanced disease stage (Table 1A) and external trigger factors as infections (Table 1B).

**Table 1:** Predisposing risk factors (A) and provoking trigger factors (B) of akinetic crisis

A. Predisposing, individual risk factors 1. Advanced disease stage (Hoehn and Yahr > 3) 2. Hallucinations, dementia 3. On–off fluctuations 4. Probably genetic Parkinsonian syndromes with mutations associated with mitochondrial dysfunction such as POLG or PINK1 mutations
B. Trigger factors 1. Infections as respiratory or urological infections 2. Premenstrual periods 3. Diabetic derailment 4. Hyponatremia 5. Trauma due to falls and bone fractures 6. Postoperative complications after surgery 7. Acute bleeding anemia 8. Hot weather and dehydration 9. Use of compound antipyretics (with pseudoephedrine, dextromethorphan) antiemetics or lithium, antidopaminergic drugs such as risperidone, amisulpride or antiemetics 10. Abrupt discontinuation of amantadine 11. Change or discontinuation of dopaminergic medication, or gastrointestinal infections or ileus, which jeopardize the absorption of dopaminergic medication

*Additional instrumental diagnostics* (Table 2) revealed elevated creatine kinase (CK) and myoglobin in serum (80%) and urine [4] in 80–100% of patients with akinetic crisis. Furthermore, elevated urea, liver enzymes, LDH, elevated leukocytes and CRP have been described even without the presence of a simultaneous infection [6].

**Table 2:** Diagnostic recommendations on akinetic crisis in PD

I. The most important recommendations:
Diagnostic criteria of akinetic crisis in PD:
1.1. Presence of risk and trigger factors as e.g. omission of dopaminergic medication, taking unfavorable anti-dopaminergic medication, operations, infections, or seasonal factors as hot weather with dehydration
1.2. Clinical symptoms as acute worsening of PD akinetic symptoms, rigidity, fever, impaired consciousness, dysphagia, accompanying vegetative symptoms, possibly myoclonus or dystonia
1.3. The clinical course with (sometimes only partial) resolution after 2–4 weeks and a high mortality rate of 4–23%
1.4. Elevated CK and myoglobin in the laboratory
1.5. Exclusion of competing causes such as serotonergic syndrome, intracranial infection, intoxication, sepsis or thyrotoxic crisis
1.6. The presence of transient levodopa resistance of the pronounced PD symptoms of at least three days
1.7. If applicable, massively reduced DAT binding in the dopamine transporter SPECT in the acute phase

Justification of the recommendation is based on moderate evidence with two longitudinal observational studies of larger cohorts, case reports and reviews, there are no randomized controlled studies available. Delphi expert consensus 100% (strong)

Electrophysiologically, a general change was found in the EEG in less than half of the Parkinson’s patients [4]; an increase in cerebral excitability in the sense of a non-convulsive status as the cause of the disturbance of consciousness should be excluded as a competing cause. Two small case series of serial DAT scans during and after the akinetic crisis [7, 8] showed transient, almost completely, symmetrically abolished, striatal presynaptic dopamine transporter binding, which partially returned to normal after the crisis [8].

**Fig. 1:**
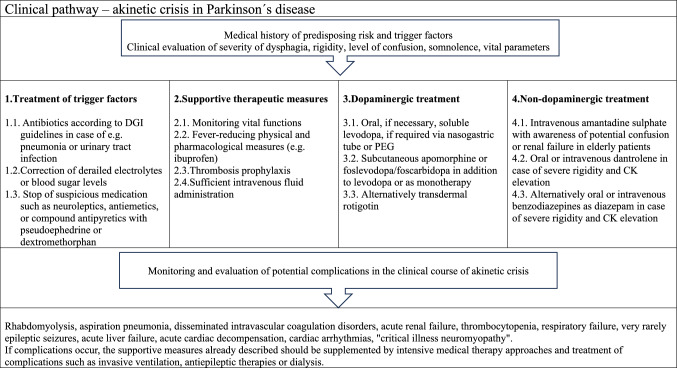
Clinical pathway of therapeutic approaches in akinetic crisis

*Therapeutic treatment* (Fig. 1, Table 3) of patients with Parkinson’s disease during the akinetic crisis poses a particular challenge [5]. Due to the pronounced dysphagia and gastrointestinal motility disorder, peroral administration of the medication is restricted. The transient levodopa resistance limits the possibilities of classic dopaminergic treatment approaches. Immobility leads to a high complication rate with intensive care treatment requirements and a high mortality rate. The adjusted and early treatment of the akinetic crisis is therefore crucial for the prognosis.

**Table 3:** Treatment recommendations on akinetic crisis in PD

II. The most important recommendations:
Treatment recommendations of akinetic crisis in PD:
2.1. Risk factors that can cause an akinetic crisis, such as infections, should be treated immediately
2.2. Supportive therapy approaches such as intravenous fluid administration, thrombosis prophylaxis, fever-reducing measures and regular monitoring of vital functions should be applied
2.3. Dopaminergic medication in the form of oral, soluble levodopa dissolved in water via a nasogastric tube, subcutaneous or sublingual application of apomorphine, transdermal rotigotine or, potentially, subcutaneous foslevodopa/foscarbidopa application should be ensured
2.4. Non-dopaminergic drugs such as intravenous amantadine sulphate or, in specific cases, dantrolene or benzodiazepines may be considered
2.5. Adequate therapy should be administered early and sufficiently, ideally in an intensive care unit, especially if complications develop

Justification of the recommendation: The evidence is based on two large, observational studies and case reports or case series without randomized controlled trials. Therapeutic approaches can be recommended from these available studies on the basis of moderate evidence. Delphi expert consensus 93.3% (strong)

In a first step, potential triggers of the akinetic crisis should be treated. Pneumonia or a urinary tract infection should be treated with antibiotics, abnormal electrolytes and blood sugar levels should be corrected, triggering medication such as neuroleptics and antiemetics should be discontinued. Second, supportive therapeutic measures as monitoring vital functions, fever-reducing medication, thrombosis prophylaxis and sufficient intravenous fluid administration should be ensured. Third, potential complications that impair the prognosis should be prevented such as rhabdomyolysis [9], aspiration pneumonia (19.2%), disseminated intravascular coagulation disorders (8.1%), acute renal failure (5.1%) [4], thrombocytopenia, respiratory failure [10], rare epileptic seizures, acute liver failure, acute cardiac decompensation (< 2%) [4] or cardiac arrhythmias.

One of the most important pillars of therapy should be the resumption and adjustment of dopaminergic medication. In the case of pronounced dysphagia, parenteral routes of administration or the use of a nasogastric or PEG tubes are recommended to ensure effective medication delivery [5]. Transient levodopa resistance must be taken into account, which should lead to adequate dose adjustment. Oral, soluble Levodopa preparations dissolved in water are most commonly used in the akinetic crisis. If dysphagia occurs, a nasogastric tube should be used for levodopa application at an early stage or a PEG tube that has already been inserted should be used. The interference of dopaminergic medication with gastroenteric nutrition must be taken into account [11], which can be prevented by predominant application of tube feeding at night and dopaminergic medication during the day, discontinuation of tube feeding 1 h before and after levodopa application or tube feeds with a lower protein content of 0.8 g/kg/day [12]. In special, treatment-resistant cases, a nasogastric levodopa-carbidopa intestinal gel (LCIG) infusion can be considered [13]. This alternative is particularly suitable if there is already an indication for PEG placement due to persistent, severe dysphagia or if LCIG therapy could also be useful after the acute crisis in the event of motor fluctuations. The newly released subcutaneous foslevodopa-carbidopa pump might be also considered, although there are no data yet available for the use in akinetic crisis. In addition to or as an alternative to levodopa, various dopamine agonists can be used in the treatment of akinetic crisis. Apomorphine can be administered via intermittent applications in the form of subcutaneous pen injections, continuously in the form of pump treatment or eventually sublingually as specific apomorphine strips [14, 15]. Apomorphine doses used in the literature are highly variable and range from 0.7 to 8 mg/h in the form of subcutaneous continuous infusion treatment and should be adjusted according to symptoms [14, 15]. Due to the frequent side effect of nausea, apomorphine should be combined with domperidone, which can also be administered rectally [16]. The use of transdermal application of the non-ergot dopamine agonist rotigotine in akinetic crisis has been described in individual cases [17–19] with a starting dose of 2 mg/24 h patch and fast increase to 6 mg/24 h within 4 days [17].

Another therapeutic approach can be the use of non-dopaminergic substances. Amantadine is particularly important as an intravenous application of amantadine sulphate in the treatment of akinetic crisis [20–23]. Amantadine appears to be particularly advantageous in the levodopa-resistant phase of the akinetic crisis due to its NMDA-antagonistic effect, as a response to NMDA antagonists may always be present here under the pathophysiological assumption of increased excitatory glutamatergic activity [22]. Amantadine doses of 200 mg to 600 mg can be used. Side effects such as delirium, confusion and renal failure must be taken into account. Administration of dantrolene has been proposed for muscle relaxation in severe rigidity in some patients during akinetic crisis [24] with symptom-oriented dosages of 1 mg/kg to a maximum of 10 mg/kg. Alternatively, the use of benzodiazepines such as diazepam to reduce muscle tone can be considered.

### The dopamine agonist withdrawal syndrome (DAWS) in Parkinson’s disease

The “dopamine agonist withdrawal syndrome (DAWS)” [25] is *defined* as a stereotypical cluster of psychiatric, autonomic and sensory symptoms that occur in temporal relation to the reduction or discontinuation of dopamine agonists and is similar to the symptoms observed in the withdrawal of psychostimulant substances (Table 4). Clinical symptoms comprise psychiatric symptoms as anxiety, panic attacks, dysphoria, depression, agitation, sleep disorders, irritability, fatigue, cravings, occasionally extending to suicidal tendencies [25]. There are also autonomic symptoms such as orthostatic hypotension, sweating, hot flushes, nausea, dizziness and generalized pain [25–29]. These symptoms do not occur in isolation, but in combination with at least 4 of the above symptoms in most patients with DAWS [26]. In the only prospective study to date, anxiety (91.7%), pain (50%), sweating (41.7%) and anhedonia (16.7%) were described as the most common symptoms of DAWS [30]. DAWS does not respond to levodopa or other PD medications except for the re-introduction of dopamine agonists [25]. This definition, sometimes referred to as the “Rabinak-Nirenberg criteria”, has not yet been validated, but has been used by all major cohort studies of DAWS and shown to be useful in practice [25–29]. According to the literature, around 15–24% of PD patients who reduce or discontinue dopamine agonists experience DAWS [25–29].

**Table 4:** Diagnostic recommendations on dopamine agonist withdrawal syndrome in PD

III. The most important recommendations:
Diagnostic criteria of dopamine agonist withdrawal syndrome (DAWS) in PD:
3.1. Presence of a severe, stereotypical cluster of physical and/or psychological symptoms
3.2. Occurrence of symptoms correlates with the withdrawal of dopamine agonists in a dose-dependent manner
3.3. Symptoms cause clinically significant impairment or social/occupational dysfunction
3.4. Symptoms do not respond to levodopa and other Parkinsonian medications, except for re-introduction of dopamine agonists
3.5. Symptoms cannot be explained by other clinical factors

Justification of the recommendation: The evidence is based on smaller, retrospective, studies, case reports or case series without randomized controlled trials, the guideline group judged the evidence to be on a weak level. Delphi expert consensus 96.4% (strong)

*Important risk factors* for DAWS represent the presence of impulse control disorders [25, 26, 31, 32], higher daily doses of dopamine agonists [25, 31–33] and higher cumulative doses of dopamine agonists [25]. In one study, the individual risk of DAWS could be estimated with knowledge of three risk factors (presence of impulse control disorders, daily dose of dopamine agonist  ≥ 150 mg levodopa equivalent dosage, previous deep brain stimulation). In this study, 92% of patients with all three risk factors developed DAWS after discontinuation of dopamine agonists, while the probability of DAWS occurring in the absence of all three risk factors was only 3% [33].

To date, *no specific therapies* are available for the treatment of DAWS [27] (Table 5). Levodopa and other dopaminergic drugs (except dopamine agonists) are ineffective [26–28, 34]. Although no controlled studies on the treatment of DAWS are available to date, existing case reports suggest that antidepressants, anxiolytics, antiepileptics, opiates and cognitive behavioral therapy have no therapeutic benefit in DAWS [25, 35]. Only the reintroduction of dopamine agonists seems to lead to an improvement in DAWS. However, as impulse control disorders often reoccur after reintroduction of the dopamine agonist, even at low doses, close monitoring is necessary in these patients [27]. There should be an individualized trade-off or weighing of DAWS and the reemergence and severity of impulse control disorders.

**Table 5:** Treatment recommendations dopamine agonist withdrawal syndrome in PD

IV. The most important recommendations:
Treatment options of dopamine agonist withdrawal syndrome (DAWS) in PD:
4.1. At present, no specific recommendation can be made on the specific treatment of dopamine agonist withdrawal syndrome due to a lack of evidence
4.2. Dopamine agonists should be considered to be discontinued slowly in order to be able to identify patients with dopamine agonist withdrawal syndromes at an early stage
4.3. In patients with severe, protracted dopamine agonist withdrawal syndrome, resumption of treatment with dopamine agonists should be considered

Justification of the recommendation: The evidence is based on retrospective studies and case reports. There is weak evidence to recommend specific treatment for DAWS. Delphi expert consensus 100% (strong)

In view of the lack of a specific therapy, several publications recommend informing PD patients and their relatives about the risk of DAWS and its symptoms before starting treatment with dopamine agonists and before discontinuing it [27, 30, 34].

Although it is not proven that the velocity of DA withdrawal plays a key role in the pathogenesis of DAWS, it is still proposed to reduce dopamine agonists slowly. Potential reduction tables suggest the reduction of piribedil 50 mg every third day, pramipexole 0,375 mg/d every day, ropinirole 2 mg every day or rotigotine 2 mg on a daily basis. Besides, one needs to consider the prognosis of DAWS, since DAWS is usually not as life-threatening as an akinetic crisis and in some PD patients, DAWS might resolve spontaneously. The long-term prognosis seems to correlate with the cumulative DA exposure [25]. In one cohort, recovery from DAWS was observed in less than 6 months in 61% of patients, in more than a year in 23% of patients, but an inability to discontinue DA in 15% of patients [26]. Thus, in the weighing procedure of risks of DAWS vs. reemerging impulse control disorder, one needs to consider that DAWS can resolve spontaneously in some patients.

If DA reintroduction is necessary, it is currently not clear, at which dosage the DA should be reintroduced. In case of severe DA induced side effects as impulse control disorder, it might be reasonable to start with low DA dosage, with the aim of finding the lowest possible dosage to resolve DAWS without crossing the threshold to reinduce impulse control disorders.

### Deep brain stimulation withdrawal syndrome (DBS-WS) in Parkinson’s disease

Deep brain stimulation withdrawal syndrome (DBS-WS) is *defined* as a potentially life-threatening condition with acute increase in akinesia and other PD cardinal symptoms, autonomic failure, fever and serum CK elevation, which occurs within hours to days after pacemaker failure in PD patients with long-standing DBS [3]. Other characteristics include the lack of response to dopaminergic medication adjustment alone, the need for hospitalization, and restitution after the earliest possible resumption of effective DBS [36, 37] (Table 6).

**Table 6:** Diagnostic recommendations on Deep Brain Stimulation withdrawal syndrome in PD

V. The most important recommendations:
Diagnostic criteria of Deep Brain Stimulation withdrawal syndrome (DBS-WS) in PD:
5.1. Presence of patient-related or technical risk factors associated with the DBS system
5.2. Clinical symptomatology of acute worsening of PD symptoms accompanied by fever, vigilance disorder, vegetative symptoms and CK elevation
5.3. Failure of DBS system that has been implanted for many years and was previously symptomatically effective
5.4. Clinical course with the presence of transient levodopa resistance of pronounced symptoms and restitution after resumption of effective DBS

Justification of the recommendation: The evidence is based on weak evidence with few case reports or case series without randomized, controlled studies. Delphi expert consensus 100% (strong)

Patient-related *risk factors* for DBS-WS are 1. young age at first manifestation of the disease, 2. bilateral implantation in the subthalamic nucleus (STN), 3. longer implantation period > 5 years, 4. advanced stage of the disease > 15 years, 5. older age and 6. excellent response to DBS with postoperative low-dose dopaminergic therapy [37–39]. However, it should be noted that in the few cases described, these initial observations need to be verified in further prospective studies of larger cohorts.

The *trigger* for DBS-WS is the abrupt failure of the DBS system, which can be caused by various factors. With rechargeable pacemaker systems, regular recharging procedures by the patient over 15-25 min/day or 90 min/week are mandatory [40], which are sometimes unintentionally omitted or inefficiently performed in elderly patients with cognitive impairment, so that the storage battery can run empty and the DBS can be switched off [41]. Battery-powered DBS systems carry an increased risk of infection of up to 5–15% [40, 42] due to the need for repeated surgical procedures. In case of hardware infection, there is the need for explantation of individual components or even the entire DBS system and a stimulation break of 6 weeks to 3 months [43, 44]. Structural care aspects such as long travel distances for the patient to DBS centers or financial aspects of unclear cost coverage or lack of regular battery checks can cause a delayed surgical stimulator change. The COVID pandemic posed a particular challenge in the provision of DBS; the sudden “running out” of batteries due to postponed or canceled outpatient check-up appointments and the cancellation of operations led to supply bottlenecks with the risk of DBS-WS [38, 45].

The *clinical course* was described in a monocentric, retrospective study of 434 DBS patients, with 15 DBS patients (3.5%) undergoing transient explantation of components of the DBS system due to an infection and stimulation break over 2–3 months. 12 patients were able to spend the waiting period at home until reimplantation despite severe motor fluctuations and motor deterioration. In 3 patients, however, DBS-WS occurred with the need for intensive care unit treatment and with a lethal outcome in 2 patients [36].

The *treatment* of Parkinson’s patients during deep brain stimulation withdrawal syndrome (DBS-WS) poses a particular challenge when stimulation breaks are unavoidable, e.g. in the case of infection-related explantation of DBS components (Table 7).

**Table 7:** Treatment recommendations on Deep Brain Stimulation withdrawal syndrome in PD

VI. The most important recommendations:
Treatment options of Deep Brain Stimulation withdrawal syndrome (DBS-WS) in PD:
6.1. Prevention of DBS-WS through close monitoring of battery status, depending on the postoperative interval, every 3–6 months and identification of patients at particular risk of possible DBS-WS
6.2. Early resumption of effective DBS, possibly through early re-implantation
6.3. If DBS stimulation pause is unavoidable, transient, drug-based, bridging therapy approaches equivalent to those used in the akinetic crisis can be considered, such as supportive therapeutic measures and high-dose administration of L-dopa, i.v. amantadine, s.c. apomorphine pump, LCIG pump or potentially s.c. foslevodopa/foscarbidopa

Justification of the recommendation: The evidence is based on weak evidence with few case reports or case series without randomized, controlled studies. Delphi expert consensus 100% (strong)

The main therapy for DBS-WS in Parkinson’s patients is the rapid resumption of effective DBS and, if necessary, early reimplantation of a pulse generator or other DBS components. Transient treatment during an unavoidable pause in stimulation, e.g. in the context of an infection of a DBS system component, is based on the treatment guidelines for the akinetic crisis (see above). In the DBS-WS literature, oral, soluble preparations of high-dose levodopa dissolved in water (up to 5 × initial dose), intravenously administered amantadine sulphate as well as pramipexole and motilium were administered via a gastric tube [46]. Furthermore, subcutaneous apomorphine pumps or LCIG pumps were used. Potentially, s.c. foslevodopa/foscarbidopa could be also used, although there are not yet data available. It should be noted that in a cohort of patients with DBS-WS, motor mobility continued to deteriorate to Hoehn&Yahr stage 5 despite increasing the dopaminergic medication to > 3 g/day [37].

The best therapy is the prevention of DBS-WS by close monitoring of the battery status. Depending on the postoperative interval, it is recommended to check the battery status in the first years every 6 months, later, when the battery is draining, in shorter intervals using the patient programmer by the patient and outpatient medical check-ups. In patients with risk factors for DBS-WS, pacemaker replacement must be given high priority with a sufficient safety margin before the elective replacement indicator (ERI) is reached [45].

In patients with risk factors for the development of DBS-WS (long-term, bilateral implantation in the subthalamic nucleus (STN) > 5 years, advanced stage of disease > 15 years, advanced age and excellent response to DBS) [37–39], reimplantation or resumption of stimulation should take place immediately. In predisposed PD patients, it has been described that despite a significant increase in the dopaminergic dose and the classic therapeutic approaches used in the akinetic crisis, no symptom improvement could be achieved until effective DBS could be restarted [36, 37]. According to surgical standards, reimplantation is not recommended until 6 weeks to 3 months after system explantation in the case of DBS system infections [43, 44] in order to avoid the risk of the infection spreading to the new pacemaker system. However, in a case series of 5 STN-DBS patients, an “early” reimplantation of the pacemaker was performed on the contralateral side in the infraclavicular lodge under continuous antibiotic coverage after an average of 23 days due to an existing DBS-WS, as soon as the local findings and the general status of the patient allowed surgery [37]. All 5 patients survived without further serious complications and regained their independence in activities of daily living in a 1-year follow-up, none of the patients showed reinfection of the new DBS system [37]. The presence of a DBS-WS thus justifies premature reimplantation of an IPG < 6 weeks outside the neurosurgical standards.

In conclusion, all acute “withdrawal syndromes” as akinetic crisis, dopamine agonist withdrawal syndrome (DAWS) or DBS-withdrawal syndrome (DBS-WS) represent rare, but potentially severe and life-threatening conditions in PD, which should be recognized early for the immediate start of the adequate therapy. Still, the evidence for diagnostic criteria and therapeutic approaches is weak to moderate due to the lack of randomized, controlled studies in these unpredictable and rare events. Multicenter, prospective, controlled studies should be performed to optimize current diagnostic and therapeutical approaches.

## Data Availability

Not applicable.
